# The GirlStars Program: Challenges to Recruitment and Retention in a Physical Activity and Health Education Program for Adolescent Girls Living in Public Housing

**Published:** 2010-02-15

**Authors:** Lee Strunin, Maisha Douyon, Maria Chavez, Doris Bunte, C. Robert Horsburgh

**Affiliations:** Boston University School of Public Health; Brigham and Women’s Hospital, Boston, Massachusetts. At the time of this study, Ms Douyon was affiliated with the Boston University School of Public Health, Boston, Massachusetts; Boston Housing Authority, Boston, Massachusetts; Community Committee for Health Promotion, Boston, Massachusetts; Boston University School of Public Health, Boston, Massachusetts

## Abstract

**Background:**

Although physical inactivity is a concern for all adolescents, physical activity levels are especially low among minority adolescents and minimal among girls from low-income families. After-school programs can reduce high-risk behaviors and strengthen schools, families, and communities.

**Context:**

We conducted an operational research project that provided free access to a program of regular, organized physical activity combined with health education sessions for adolescent girls in 2 public housing developments in Boston, Massachusetts.

**Methods:**

From July 2002 through October 2005, at each of 2 public housing sites, the GirlStars program participants met each week for two 2-hour sessions, 1 dedicated to physical activity and 1 dedicated to health education. Sessions were led by the project coordinator and a resident assistant at each development.

**Outcome:**

Participants in the GirlStars program increased their health knowledge, self-confidence, and decision-making skills, but rates of participation were low. Factors that affected participation included safety concerns, lack of community support for the program, interpersonal conflicts, attrition in staff, and conflicts with other activities.

**Interpretation:**

Programs in public housing developments that address these barriers to recruitment and retention may be more successful and reach more girls.

## Background

Racial/ethnic minority adolescents are less active than are white adolescents, and girls from low-income families are particularly inactive (1-4). The prevalence of obesity is twice as high in black girls as in white girls (3). One study of teenage girls found that factors such as safety, privacy, and cultural issues contribute to low rates of participation in physical activity outside school (5).

After-school programs can improve adolescents' behavior, performance, and health (6). These programs may reduce high-risk alcohol and drug use, reduce juvenile crime, enhance children's academic achievement, support children's social development and their relationships with adults and peers, and strengthen schools, families, and communities (7). Participating in physical activity programs enhances physical, emotional, and social aspects of health (8). A study of African American and Latina middle school girls found that fun activities, body image concerns, and social support motivated them to be physically active (9). Other studies indicate a need for sex-specific interventions in comfortable settings; these interventions should use activities that the girls help plan (10). Traditional physical education classes, however, are often not enjoyable for girls and discourage those who may be interested in sports or other physical activity (11).

## Context

We present findings from a program designed to address physical activity and health education needs among inner-city adolescent girls living in public housing developments in Boston, Massachusetts. The GirlStars program was the core research project of the Partners in Health and Housing Prevention Research Center (PHH-PRC). The PHH-PRC is an equal partnership among Boston University School of Public Health, Boston Housing Authority (BHA), Boston Public Health Commission, and the Community Committee for Health Promotion, which is composed of public housing residents and community advocates. The mission of the PHH-PRC is to engage public housing residents in community-centered research programs and activities that improve their health and well-being.

BHA is a public agency that provides subsidized housing to low- and moderate-income people. BHA is the largest landlord in Boston and the largest public housing authority in New England. The agency houses approximately 10% of the city's residents. Approximately 80% of these households have annual incomes less than $15,000. Among the residents, 36% are Hispanic, 31% are non-Hispanic black, 22% are non-Hispanic white, and 8% are Asian. Nearly 40% of residents are children. The agency's housing developments are concentrated in neighborhoods of Boston with the highest incidence of indicators of poor health, including injuries and environment-related illnesses such as asthma, lead poisoning, and smoking.

## Methods

We designed the GirlStars program to provide adolescent girls aged 9 to 13 years who live in Boston public housing developments regular, organized physical activity combined with health education. The goal of the intervention was to promote a lifetime of physical activity and positive health behaviors. This study was approved by the institutional review board at Boston University Medical Center.

From July 2002 through October 2005, the intervention was conducted at 2 housing developments in Boston. Group meetings were held in a community meeting space at each housing development for 2 hours after school, twice per week; meetings were often held outside, weather permitting. One meeting session was dedicated to health education and the other focused on physical activity. Both sessions were led by the GirlStars project coordinator and program assistants. Girls provided input for health education and physical activity sessions at the beginning of the program, and many of their suggestions were incorporated into the curriculum.

Health education sessions focused on improving knowledge of risk behavior and increasing self-efficacy, resilience, and self-confidence. Discussion topics included body image, goal setting, self-esteem, development, nutrition, and healthy eating. All sessions included practical activities and group work that involved the girls.

Girls completed a physical activity and behavior questionnaire at enrollment and at the end of the program. Height and weight were also measured at these times.

The program recruitment goal was 80 girls, 40 from each of 2 housing developments, with an intervention and comparison group. Because of low rates of participation, however, all the girls were enrolled in the intervention group. Sites were selected on the basis of the number of eligible girls living there and presence of experienced on-site youth workers who would assist with recruitment and programming. When the program was planned, BHA employed youth workers who conducted youth programming at each housing development.

GirlStars program staff used recruitment tools and approaches at both sites, including flyers, open houses, mailings, attending community events, knocking on the doors of all households with eligible girls, and face-to-face contact. BHA staff also assisted with targeted mailings to appropriate girls.

## Outcome

During the 3 years of the program, 60 girls enrolled. Some girls enrolled at baseline and continued throughout the study; others attended periodically, moved from the development, dropped out, or aged out of the program. Of the 33 participants enrolled at baseline, 31 agreed to be measured. At baseline, 6 of 14 (43%) girls at site A were overweight, compared with 4 of 17 (24%) girls at site B. All 33 girls completed the physical activity questionnaire. Responses indicated that girls at site A were less likely than girls at site B to have participated in physical activity for at least 20 minutes per day in the week before the questionnaire. By the end of the program, girls at both sites increased their physical activity level and improved their knowledge of nutrition and healthy eating.

At the end of the program, 10 girls at site B participated in individual, semistructured, open-ended interviews, during which the girls were asked about their reasons for joining the program, reasons for attending group sessions, benefits of participation, and recommendations for future programs. Responses reinforced the need for after-school programming. Being bored and having "nothing to do" were common reasons for participating in the program. Girls reported that they learned about personal health and had increased self-confidence in decision making. They also reported that the supportive environment, which was fostered by the program leader's ability to connect with girls and make them feel comfortable talking freely, contributed to the positive outcomes of the program.

A number of challenges affected recruitment and participation, including program location and safety, interpersonal conflicts, reluctance to participate in physical activity, lack of community support, lack of continuity in staffing, and conflicts with other activities.

### Recruitment

Recruiting girls to the program was the biggest challenge. When the program began, BHA lost funding for its youth workers. This change in staffing directly affected the program, since GirlStars program staff had to devote more of their time to recruitment. GirlStars staff, unfamiliar with the housing developments and residents and often considered "outsiders," had difficulty identifying and recruiting participants. After the youth workers left, we saw that to build community interest in the program, we needed someone from the development or known by residents. We intensified efforts to recruit program assistants who were residents of the development to help lead group sessions and with recruitment and retention.

At site A, a local community center referred girls to the program, but because the community center was close to site A, community center activities competed with GirlStars activities, which resulted in irregular attendance. At site B, a BHA staff member was a resident services coordinator, and she identified and recruited potential participants and program assistants. She worked as a liaison with parents and property management in the absence of a program assistant.

### Program location and safety

At each development, the program was located at former youth centers. Although these centers were centrally located, feedback indicated that parents were concerned about their daughters' traveling to other parts of the development to attend the program.

At site A, the meeting space was vandalized several times, and other youths at the development harassed and threatened program participants, which decreased participation. The program was relocated to another space in the development, but the harassment continued. To address this problem, a uniformed officer was present during group meetings for a time. Additionally, the program coordinator and the BHA resident services coordinator defined rules of behavior and consequences to reestablish control over the group. Some girls were expelled from the group until they could demonstrate the ability to behave and conform to group expectations.

### Interpersonal conflicts

In addition to the safety of the girls, personal conflicts directly affected participation. Dynamics among girls and cliques prevented some girls from feeling comfortable attending meetings. At site A, several participants were suspended for physically fighting. Some parents did not want their daughters to participate because of other participants.

### Reluctance to participate in physical activity

Girls' reluctance to participate in the physical activity portion of the program was a challenge that required creativity in gaining the girls' interest. Other research on adolescent girls and physical activity found that participants associate physical activity with exercise and sports and do not see it as fun (10). Dispelling myths about physical activity and making these activities fun for girls motivated their participation.

### Community support

For community involvement to be successful, researchers and the community must be partners in decision making (9). Eliciting community support was among the biggest challenges for the program. When the program was implemented, neither site had a tenant organization that could promote or advise the program. To build community interest in the program, we needed someone from the development and known by residents. At each development, on-site property managers introduced us to the development and provided logistic support, such as securing space. On occasion, managers identified eligible girls and referred participants. At each site, managers and office staff were informed about the program and asked to pass along information to households with eligible girls. The involvement of management and other development staff was essential to the program.

### Staffing

At both sites, difficulties hiring and retaining resident assistants resulted in frequent turnover, which compromised program stability. Reasons for not remaining in the position included the need for full-time employment, lack of experience, and medical issues. At both sites, a program assistant was eventually hired and remained with the program until its conclusion. The program assistant provided continuity for the girls, provided ongoing support, and was integral to involving parents with the program. Because the program assistant was a resident of the development, she offered insight into the concerns of participants' parents and other residents and was a connection to the program outside group meetings.

### Conflicts with other activities

As in other studies (11), the most frequent explanation for not participating in physical activity was conflict with other activities. Family responsibilities, including caring for younger siblings, and extracurricular activities prevented girls from participating in our program. Among African American and Latina girls, another barrier to engaging in physical activity is appearance and self-image (9,10). The need for social support and the influence of attitudes and behaviors of friends often determine whether girls exercise (9,10). Many girls participated in our program because their friends were participating, but our girls, like others, did not see exercise and sports as "fun."

## Interpretation

Although we did not meet our recruitment goals and the small number of participants prevents us from drawing firm conclusions, we believe that the GirlStars Program was beneficial to the girls who participated. The girls were involved in the planning of health education and physical activity sessions, which may have encouraged their participation. They also reported that their relationships with other girls and with the program staff motivated them to remain in the program. Program sessions focused on increasing self-efficacy, resilience, and self-confidence, and participants had increased health knowledge, self-confidence, and decision-making skills and became more physically active. The length of time needed for such a program to become established might vary in different contexts. The positive reinforcements from the program encouraged participation, but time is needed to nurture the personal relationships that lead to bonding between participants and staff. For a program to be successful in public housing developments, broad community support is essential. In addition, awareness of underlying factors that may affect participation and the ability to design programs that address those needs are vital for any program in public housing. A number of lessons from this program may be applicable to other organizations seeking to enroll girls in a program in public housing developments or other urban, multicultural, low-income populations.

### Site selection

Sites should be selected after a comprehensive evaluation process. For future programs, the Prevention Research Center developed criteria on the basis of a review and assessment of the first 2 years of GirlStars. The Prevention Research Center has created a document that will be distributed among all public housing developments in Boston to engage tenant organizations, property managers, and community agencies for future programs. An organizational stipend will be available to tenant organizations for the assistance they provide to the program. The document also assesses types of programming the target population would be interested in and level of community support for the program.

### Participant recruitment and retention

Local tenant organizations, resident assistants, and managers at the developments must be involved in recruiting participants. Buy-in from the community is needed for program success. The local tenant organization must be used in outreach to households and in identifying girls. The development should also partner with other youth-serving agencies to attract girls from the development.

To maximize participation in programs, tenant organizations should also be involved in decisions regarding the schedule of the program. Participants enrolling in the program should be consulted about activities and topics to be discussed in meetings. Group leaders should try to incorporate the desires of participants into program curricula.

### Other lessons learned

Physical safety was a major concern of our participants and their parents. The level of violence in these housing developments and their surrounding neighborhoods is high, and safety concerns must be mitigated by careful choice of program location, hours, and supervision.

A resident assistant must be hired at each development to assist in program activities and in recruiting and retaining participants. Resident assistants must also receive training to build their skills in working with the target population. They should maintain a close working relationship with the tenant organization and management staff of the housing development.

A program-specific advisory board must also be established at each development; this board should consist of interested parents and other residents, the tenant organization, management staff of the housing development, and members of other community organizations. The advisory board should provide oversight for the program and advise program staff about the curriculum and program activities. The advisory board must also communicate with program staff the needs and issues in the developments as they relate to the program and its participants.

Attaining the support of management from each housing development, beginning at program inception, is essential to success. Managers and other staff can help with problems and build support for the program among residents. Management at each development must become familiar with the program and its components to provide information to new residents and help identify and recruit participants.

### Conclusions

Participants in the GirlStars program increased their knowledge of personal health, self-confidence, and decision-making skills; however, the program did not reach many girls. The major barriers to participation were safety concerns, interpersonal conflicts, reluctance to participate in physical activity, lack of community support, lack of continuity in staffing, and conflicts with other activities. Future programs that address these barriers may be more successful and reach more girls.
